# Bromide of Lithia

**Published:** 1890-04

**Authors:** 


### Bromide of Lithia.

Lithia and its salts have a desirable restraining action on any tendency to excessive destructive metamorphosis within the system. They prevent excessive phosphatic waste, thus sustaining the nervous system; they retard, to a certain extent, the tendency to rapid conversion of the nitrogenous food elements into urea, quite common among brain workers--business and professional men, especially, whose minds are weighed down with anxiety and care. They retard the excessive formation, also, of uric acid, and are thus of great value in a wide range of diseases. Combined with bromine, it is quite evident that the therapeutic effects of the remedy would be most desirable. It is thus especially adapted to those cases of nervous excitement which follow nerve exhaustion; nervous irritation and insomnia from overwork, or in all nerve irritations that follow starved nerve conditions. The bromide of lithia may be given in all the conditions mentioned, and in those where the sodium or potassium salt are indicated, with excellent results. The dose is from five to thirty grains. In epilepsy its influence is mild but effectual and permanent, even more satisfactory than the other bromides. In threatened apoplexy it will quickly soothe the patient and ward off an attack. In acute attacks of cerebral hyperæmia, with intense headache and nervous excitement, it works admirably and quickly. In these cases it may be well to combine it with ergot in appropriate doses.

In all cases of overwork of the nervous system, followed by the long train of symptoms of nervous prostration, the bromide of lithia should be given in combination with the restorative agents and tonics.

Its field is a wide one, and should be better known and more often prescribed.—*Chicago Med. Times.*

